# Fear renewal preferentially activates ventral hippocampal neurons projecting to both amygdala and prefrontal cortex in rats

**DOI:** 10.1038/srep08388

**Published:** 2015-02-11

**Authors:** Jingji Jin, Stephen Maren

**Affiliations:** 1 Department of Psychology and Institute for Neuroscience, Texas A&M University, College Station, Texas 77843 USA

## Abstract

Anxiety, trauma and stress-related disorders are often characterized by a loss of context-appropriate emotional responding. The contextual retrieval of emotional memory involves hippocampal projections to the medial prefrontal cortex and amygdala; however the relative contribution of these projections is unclear. To address this question, we characterized retrieval-induced Fos expression in ventral hippocampal (VH) neurons projecting to the prelimbic cortex (PL) and basal amygdala (BA) after the extinction of conditioned fear in rats. After extinction, freezing behavior (an index of learned fear) to the auditory conditioned stimulus was suppressed in the extinction context, but was “renewed” in another context. Hippocampal neurons projecting to *either* PL or BA exhibited similar degrees of context-dependent Fos expression; there were more Fos-positive neurons in each area after the renewal, as opposed, to suppression of fear. Importantly, however, VH neurons projecting to *both* PL and BA were more likely to express Fos during fear renewal than neurons projecting to either PL or BA alone. These data suggest that although projections from the hippocampus to PL and BA are similarly involved in the contextual retrieval of emotional memories, VH neurons with collaterals to both areas may be particularly important for synchronizing prefrontal-amygdala circuits during fear renewal.

In recent years, considerable effort has been focused on understanding the neural mechanisms underlying the extinction and renewal of learned fear^1^,^2^,^3^,^4^,^5^,^6^. During extinction, repeated exposure to an aversive conditioned stimulus (CS) gradually decreases the probability and magnitude of the conditioned fear response (CR)^7^,^8^. However, substantial evidence suggests that extinction does not eliminate the fear memory; rather, it generates a new extinction memory that competes with the fear memory for control of behavior^9^,^10^. Importantly, the extinction memory is highly context-dependent insofar as it is only expressed in the extinction context. That is, if animals encounter the CS outside of the extinction context, the conditioned fear response returns or “renews”. The renewal of extinguished fear is a considerable challenge for maintaining long-lasting fear suppression after exposure-based therapies for anxiety, trauma, and stress-related disorders^11^,^12^,^13^.

Recent studies reveal that a brain circuit involving the hippocampus, medial prefrontal cortex (mPFC) and basal amygdala (BA; including the basolateral and basomedial nuclei) is important for the context-dependence of extinguished fear memories^14^,^15^,^16^,^17^,^18^. For example, pharmacological inactivation of the hippocampus disrupts context-dependent firing in the amygdala^19^and prevents fear renewal^20^. Inactivation of the ventral hippocampus regulates the expression of spike firing in the prelimbic (PL) region of the mPFC^21^and impairs fear renewal^22^. Furthermore, immediate early gene expression in the VH, BA and PL is context-dependent^15^,^16^and amygdala neurons receiving PL and VH efferents are recruited during fear renewal^18^. Lastly, disconnection of VH inputs to either the BA or PL eliminates fear renewal^17^. These findings suggest that the hippocampus may gate neural activity in either the mPFC or BA to regulate the context-dependent expression of fear after extinction^23^. However, the relative contribution of VH projections to the BA and mPFC in this process is not clear.

Within the ventral hippocampus, neuroanatomical studies have shown that the projections to the BA and mPFC originate from ventral CA1 (vCA1) and the ventral subiculum (vSUB)^24^,^25^,^26^. Although the majority of vCA1 and vSUB neurons project to either the BA or mPFC, some VH neurons project to both areas^27^. These “dual-projecting” neurons (i.e., VH neurons projecting to both PL and BA) may be particularly important for coordinating mPFC and BA activity during memory retrieval, a function suggested by the similar consequences of VH-PL and VH-BA disconnections on fear renewal^17^. To explore this question, we used fluorescently labeled retrograde tracers (AlexaFluor-conjugated cholera toxin B, CTb) and c-Fos immunohistochemistry to quantify retrieval-related Fos expression in vCA1 and vSUB neurons projecting to the PL and/or BA. Our results suggest that dual-projecting neurons in vCA1 and vSUB play a particularly important role in the contextual retrieval of fear memories after extinction.

## Results

**Figure 1:**
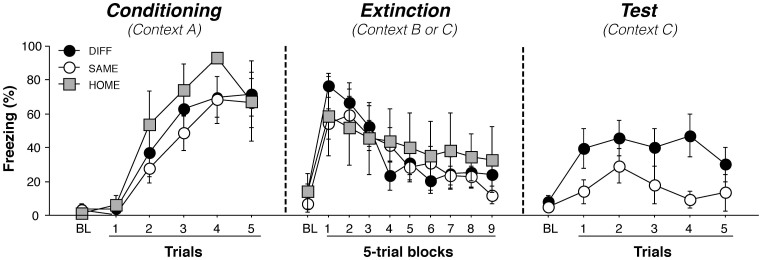
Conditioned freezing behavior. (*A*), Mean (±SEM) percentage of freezing during fear conditioning. Freezing was averaged across the 3-min pre-CS baseline (BL) as well as during each of the five conditioning trials; each trial consisted of the average of freezing during each CS presentation and the subsequent ITI. (*B*), Mean (±SEM) percentage of freezing during the 45 tone-alone extinction session. Freezing was averaged across the BL period as well as during the 45 extinction trials; as with conditioning, each trial consisted of the average of freezing during each CS presentation and subsequent ITI (data are presented as 9 five-trial blocks). (*C*), Mean (±SEM) percentage of freezing during the test session, which consisted of five tone-alone presentations with 30 s ITIs. Freezing was measured during the BL period and during the five trials, each of which consisted of a CS presentation and the subsequent ITI. Data are shown for rats that were tested outside the extinction context (DIFF; black circles), tested within the extinction context (SAME; white circles), or not tested at all (HOME; gray squares).

Freezing behavior during the conditioning session is shown in Figure 1. All rats increased their levels of freezing during the conditioning session [main effect of block, *F*(5, 95) = 32.4, *p* < 0.001] and the levels of freezing did not differ between the groups [main effect of group and group × block interaction, *F*s < 1]. During extinction (Figure 1, middle panel), rats in all of the groups exhibited high levels of conditioned freezing to the CS and similar reductions in conditioned freezing across the extinction session [main effect of block, *F*(9, 171) = 15.3, *p* < 0.001; main effect of group and group × block interaction, *Fs* < 1.3]. As many previous studies have shown, the expression of conditioned freezing to the extinguished CS was context-dependent (Figure 1, right panel). Conditioned freezing was low when the extinguished CS was presented in the extinction context (SAME), whereas rats tested outside of the extinction context (DIFF) exhibited higher levels of conditioned freezing (Figure 1, right panel) [main effect of group, *F*(1,14) = 4.5, *p* < 0.05]. Importantly, renewal was not attributable to the contextual freezing because baseline freezing to the context was not significantly different between groups (*p* = 0.4). Moreover, differential freezing among the SAME and DIFF groups was not attributable to physical differences in the test contexts since all testing was conducted in an identical context with the same CS.

**Figure 2:**
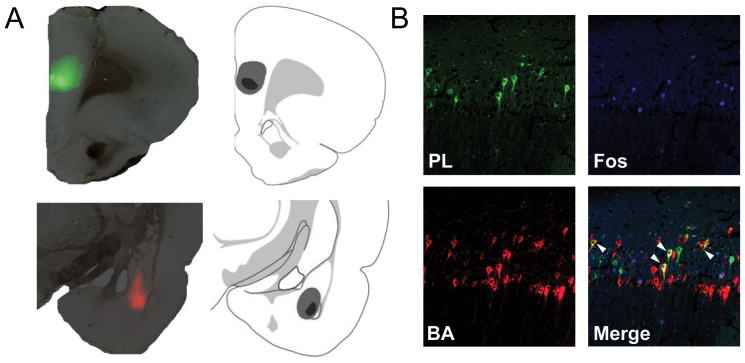
Alexa fluor conjugated cholera toxin B (CTb) infusion sites within the PL and BA. (*A*), Representative coronal sections displaying the site of the CTb injections in PL and BA; schematic indicates maximal (gray) and minimal (black) spread in each region. (*B*), Photomicrographs of representative coronal sections (10×) showing Fos- and CTb-labeling in rats from a representative rat. BA-projecting neurons are red, PL-projecting neurons are green, and dual-projecting neurons (PL + BA, white arrowheads) are yellow; Fos-positive neurons are blue.

**Figure 3:**
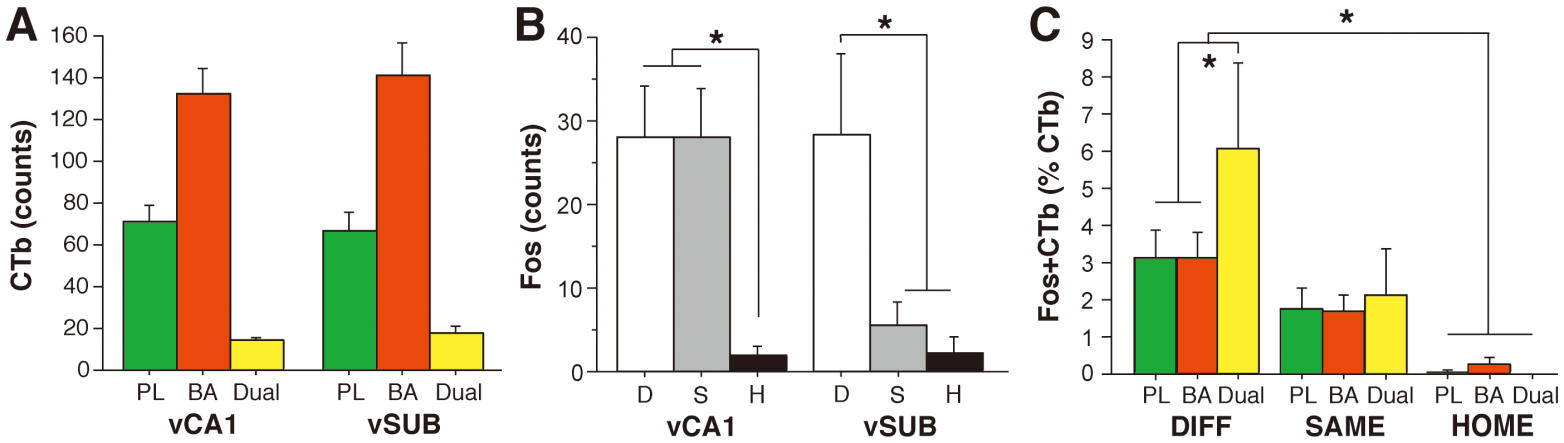
Quantification of CTb labeling and Fos expression in neurons in the vCA1 and vSUB after fear renewal. (*A*), Mean (±SEM) cell counts for CTb-positive neurons in vCA1 and vSUB collapsed across retrieval condition. Neurons in both vCA1 and vSUB projected to the prelimbic cortex (PL, green), basal amygdala (BA, red), or both areas (Dual, yellow). (*B*), Mean (±SEM) cell counts for Fos-positive neurons in vCA1 and vSUB among animals tested outside the extinction context (DIFF, D), inside the extinction context (SAME, S), or untested animals (HOME, H). (*C*), Mean (±SEM) percentage of Fos-positive projection neurons (PL, BA, or dual-projecting Fos- and CTb-positive neurons divided by CTb counts alone) in the vCA1 and vSUB of animals in each of the behavioral groups.

Ninety minutes after retrieval testing, rats were perfused with paraformaldehyde and their brains were extracted. Representative CTb injection sites in PL and BA are shown in Figure 2A along with a schematic illustration of maximal and minimal infusions. PL- and BA-projecting neurons in vCA1 and vSUB were labeled with different AlexaFluor-CTb conjugates and c-Fos was visualized with AlexaFluor 350 (Figure 2B). For all of the animals in the analysis, the number of CTb-labeled neurons in vCA1 and vSUB are shown in Figure 3A. In both hippocampal regions, there were more neurons projecting to BA than PL, and a significantly smaller proportion of the neurons projecting to both areas. This impression was confirmed by a two-way ANOVA with factors of brain region (vCA1 and vSUB) and cell type (PL, BA or PL + BA) that revealed a main effect of neuron type [*F*(2,6) = 113; *p* < 0.001]. Post hoc comparisons revealed significantly higher number of BA-projecting neurons than PL-projecting neurons in both vCA1 and vSUB (*p* < 0.0001). In addition, the number of dual-projecting neurons was significantly lower than the numbers of neurons projecting to either PL or BA alone (*p* < 0.0001). Dual-projecting neurons accounted for roughly 10% of the total labeled neurons in the ventral hippocampus.

We next examined Fos expression in all vCA1 and vSUB neurons (regardless of their projection targets) as a function of memory retrieval group (SAME, DIFF, or HOME). As shown in Figure 3B, Fos expression in vCA1 and vSUB was influenced by the context in which retrieval testing occurred. In vCA1, the number of Fos-positive neurons in both of the retrieval conditions was similarly elevated relative to HOME controls. However, Fos expression was context-dependent in vSUB. That is, Fos expression in vSUB was greater when the CS was presented outside the extinction context (DIFF) relative to when it was presented in the extinction context (SAME) or in rats that were not tested (HOME). These impressions were confirmed in a two-way ANOVA with factors of brain region (vCA1 and vSUB) and group (DIFF, SAME, and HOME) that revealed a significant main effect of group [*F*(2,17) = 4.52, *p* < 0.05] and a significant group X region interaction [*F*(2,17) = 4.47, *p* < 0.05]. Post-hoc comparisons (*p* < 0.05) revealed that in vCA1 both SAME and DIFF rats exhibited greater levels of Fos expression than rats in the HOME control (but did not differ from one another). In contrast, in vSUB rats in the DIFF condition exhibited significantly greater Fos expression than rats in the SAME or HOME groups (which did not differ from each other).

We next examined whether Fos was expressed in VH neurons projecting to PL, BA, or both areas, and whether neurons with different efferent targets exhibited different degrees of Fos expression. In general, only a small percentage (~5%) of CTb-labeled neurons expressed Fos in the retrieval conditions, a finding that is consistent with previous reports^17^. A three-way ANOVA with factors of group (SAME, DIFF or HOME), brain region (vCA1 or vSUB) and cell-type (PL-, BA-, or dual-projecting) revealed only a significant main effect of group [main effect of group, *F*(2,18) = 4.26, *p* < 0.05]; all other main effects and interactions fell short of significance. Because the pattern of Fos expression in CTb-labeled neurons did not differ between vCA1 and vSUB, we collapsed the neurons in these areas to simplify the analysis. As shown in Figure 3C, Fos expression among CTb-labeled neurons was highest among rats in the DIFF group. Post-hoc comparisons (*p* < 0.05) revealed that DIFF rats (the condition associated with fear renewal) exhibited a greater percentage of Fos-positive CTb-labeled neurons than rats in the HOME control; SAME rats did not differ from the HOME control. These results are congruent with previous results from our laboratory showing renewal-induced increases in Fos expression in BA-projecting neurons in the ventral hippocampus^17^.

Importantly, inspection of Figure 3C reveals that a greater proportion of dual-projecting neurons exhibited Fos expression relative to neurons projecting only to BA or PL when animals renewed their fear (i.e., in the DIFF condition). Planned comparisons in the form of one-way ANOVAs in each behavioral group revealed a significant reliable between the cell types in only the DIFF condition [*F*(2, 14) = 3.70, *p* < .05]. Post-hoc comparisons (*p* < 0.05) revealed that dual-projecting neurons were nearly twice as likely to express Fos as neurons projecting to either PL or BA alone. Hence, among the entire population of vCA1 and vSUB neurons that projected to BA or PL, the subset of neurons projecting to both regions (i.e., the dual-projecting neurons) was more likely to express Fos after fear renewal.

**Figure 4:**
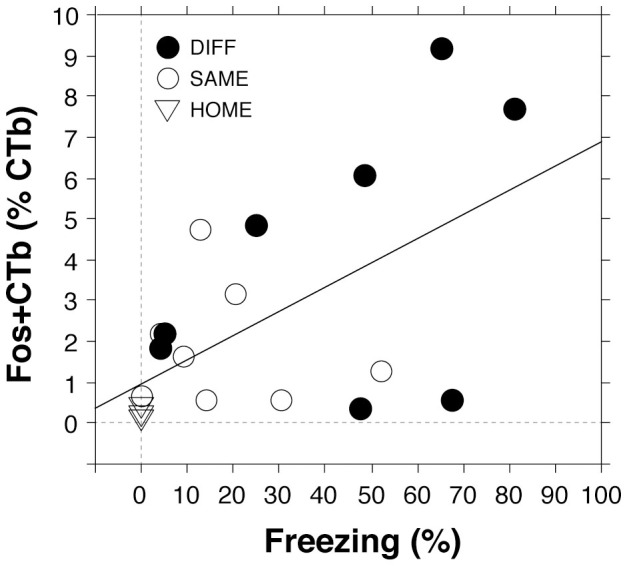
Correlation between the number of Fos-positive CTb-labeled cells in the vCA1 and vSUB with average freezing behavior during the retrieval test among rats in DIFF, SAME, and HOME groups.

The present results reveal that the renewal of extinguished fear is associated with Fos expression in vCA1 and vSUB neurons projecting to PL and/or BA. We were interested in determining whether there was a relationship between the number of Fos-positive projection neurons in the ventral hippocampus and freezing behavior during the retrieval test. As shown in Figure 4, there was a strong positive correlation (Pearson *r* = 0.57, *p* < .01) between freezing behavior and the number of Fos-positive projection neurons when the rats in each group were aggregated. This supports the view that the ventral hippocampus plays a key role in fear renewal, and suggests that relapse may be particularly dependent on those neurons projecting to the prefrontal cortex and basal amygdala.

## Discussion

The present data reveal that the retrieval of an extinguished CS induces context-dependent Fos expression in ventral hippocampal neurons projecting to the medial prefrontal cortex and basal amygdala. The context-dependence of Fos expression in the ventral hippocampus among all neurons was greatest in vSUB; Fos expression in vCA1 was not itself context-specific when all Fos-positive neurons in the region were considered. However, when neurons were parsed by their projection targets, we observed that PL- and BA-projecting neurons in both vCA1 and vSUB exhibited context-dependent Fos expression. Specifically, greater numbers of projection neurons expressed Fos in the renewal condition (DIFF test) compared to the extinction condition (SAME test). Interestingly, although few in number, dual-projecting neurons with collaterals to both PL and BA were more likely to exhibit Fos expression than neurons projecting only to PL or BA after fear renewal. The greater proportion of dual-projecting neurons expressing Fos during fear renewal suggests that these cells may be particularly important for synchronizing prefrontal-amygdala circuits involved in fear expression^28^.

Previous results indicate that fear renewal increases Fos expression in the VH^15^, including in VH neurons projecting to the BA^17^. Interestingly, we now show that there are important differences in the context-specificity of Fos expression insofar as neurons in vCA1 are engaged in memory retrieval regardless of where the extinguished CS is encountered, whereas vSUB neurons are Fos-active only in the renewal condition. This suggests that vCA1 may have a general role in memory retrieval engaged by the presentation of ambiguous stimuli^29^,^30^. In contrast, vSUB neurons may be involved in an associative mismatch process that occurs when an animal encounters a familiar CS in a familiar context for the first time (i.e., a situation that promotes renewal)^31^,^32^,^33^. Indeed, the vSUB may be particularly important for integrating contextual memories retrieved by the hippocampus with emotional information retrieved by the amygdala to drive context-dependent fear behavior^25^.

The crucial role of the hippocampus in contextual memory retrieval has been shown in many previous studies^34^,^35^,^36^. After extinction, it has been suggested that the ventral hippocampus uses contextual information to “gate” the expression or suppression of fear in a given context^1^,^23^,^37^. Consistent with this view, infusion of the GABA_A_ agonist muscimol into the ventral hippocampus disrupts context-dependent fear memory retrieval outside of the extinction context^22^. Importantly, hippocampal inactivation does not affect fear expression to a non-extinguished CS or disrupt context discrimination^20^,^38^,^39^. Rather, hippocampal inactivation impairs the retrieval of CS-context associations necessary to guide context-dependent behavior.

We have previously suggested that the ventral hippocampus gates fear behavior through its projections to the basal amygdala^5^,^17^. The ventral hippocampal formation, including vCA1 and vSUB, has robust reciprocal projections with amygdala, a structure that is essential for acquisition and expression of the fear memory^4^,^40^,^41^. Muscimol infusion in the ventral hippocampus eliminates context-dependent neuronal activity in the amygdala^19^ and BA neurons that fire during fear renewal receive direct projections from ventral hippocampus^14^. Disconnection of hippocampal projections to the BA also eliminates fear renewal after extinction^17^. Moreover, in the present experiment, vCA1 and vSUB neurons projecting to BA expressed Fos after fear renewal. Collectively, these studies indicate that hippocampal projections to the amygdala are essential for context-dependent fear expression.

Another possibility is that VH projections to the mPFC regulate the expression of fear after extinction. Both the vCA1 and vSUB project strongly to the mPFC, including PL^26^,^42^, which in turn has robust connections with the amygdala^43^. Recent work indicates that disconnecting VH projections to PL impairs fear renewal^17^ and VH inactivation modulates PL spike firing and regulates the expression of extinguished fear^21^. Moreover, mPFC projections to the amygdala are important for both the expression and suppression of fear after extinction^6^,^44^,^45^,^46^. The present data are consistent with the role of VH projections to mPFC in the regulation of extinguished fear insofar as VH neurons projecting to PL exhibited high c-Fos expression after renewal.

Interestingly, we found that VH neurons with projections to both the PL and BA (dual-projecting neurons)^27^ were engaged to a greater degree than VH neurons projecting to either the PL or BA alone. These dual-projecting neurons have similar antidromic latencies to mPFC and amygdala stimulation, which suggests that spiking in VH neurons can simultaneously activate both the mPFC and the amygdala^27^. Even though the total number of dual-projecting neurons is small, our results suggest that they might play a particularly important role in fear memory retrieval after extinction. Indeed, these neurons may play an important function in synchronizing neuronal activity in the mPFC and BA to overcome extinction-related inhibition and promote conditional responding^28^,^47^,^48^,^49^. Together with previous data, the present results support the view that the contextual retrieval of emotional memories involves hippocampal coordination of neuronal activity in prefrontal-amygdala circuits that regulate fear expression^5^.

## Methods

### Subjects

The subjects were 26 Long-Evans male adult rats (200–224 g; Blue Spruce) obtained from Harlan. The rats were individually housed on a 14/10 h light/dark cycle and had access to food and water *ad libitum*. Rats were handled for 5d before the experiment. All experimental procedures were performed in accordance with the protocols approved by the Texas A&M University Animal Care and Use Committee.

### Behavioral apparatus

Eight identical observation chambers (30 × 24 × 21 cm; MED-Associates) were used in all behavioral sessions. The observation chambers were constructed of two aluminum sidewalls and a Plexiglas ceiling, rear wall, and hinged front door. The floor of each chamber consisted of 19 stainless steel rods that were wired to a shock source and a solid-state grid scrambler (MED-Associates) for the delivery of foot shock (US). A speaker mounted outside of the grating in one wall of the chamber was used for the delivery of acoustic CS. Additionally, ventilation fans and house lights were installed in each chamber to allow for the manipulation of contexts. Sensory stimuli were adjusted within these chambers to generate three distinct contexts A, B and C. For context A, a 15-W house light mounted opposite to the speaker was turned on, and the room light remained on. Ventilation fans (65 dB) were turned on, cabinet doors were left open, and the chambers were cleaned with 70% ethanol. Rats were transported to context A in white plastic boxes without beddings. For context B, house lights were turned off and overhead lighting was provided by fluorescent red lights. Ventilation fans were turned off, the cabinet doors were closed and the chambers were cleaned with 1% acetic acid. Rats were transported to context B in black plastic boxes without beddings. For context C, both the house lights and the red fluorescent overhead lights were turned on. Ventilation fans were off and the cabinet doors were left open. Black Plexiglas floors were placed on the grid of each chamber and the chambers were cleaned with 1% ammonium hydroxide. Rats were transported to context C in white buckets with beddings. In each context, stainless steel pans were filled with a thin layer of the respective odors of the contexts and inserted below the grid floor.

Each conditioning chamber rests on a load-cell platform that is used to record chamber displacement in response to each rat's motor activity and is acquired online via Threshold Activity software (MED Associates). Before the experiment, all load cell amplifiers were calibrated to a fixed chamber displacement and load-cell amplifier output (−10 to +10 V) from each chamber is digitized and absolute values of the load-cell voltages are computed and multiplied by 10 to yield a scale that ranges from 0 to 100. For each chamber, load-cell voltages are digitized at 5 Hz, yielding one observation every 200 ms. Freezing is quantified by computing the number of observations for each rat that has a value less than the freezing threshold (load-cell activity = 10). Freezing is only scored if the rat is immobile for at least 1 sec.

### Surgical procedures

Rats were anesthetized with ketamine (100 mg/kg, i.p.) and xylazine (10 mg/kg, i.p.) and given atropine sulfate (0.4 mg/kg, i.p.). After induction of anesthesia, the rats were placed into stereotaxic apparatus (David Kopf Instruments) and 27-gauge injectors were lowered into the BA [anteroposterior (AP), −3.0 mm; mediolateral (ML), ±5.1 mm; dorsoventral (DV), −9.2 mm from skull] and PL [anteroposterior (AP), +2.9 mm; mediolateral (ML), ±1.5 mm; dorsoventral (DV), −3.5 mm from dura]. For PL injection, the stereotaxic arm was lowered at a 15° angle to prevent damage to the superior sagittal sinus. The injector was attached to polyethylene tubing, which was connected to a Hamilton syringe (10 μl) located on an infusion pump. AlexaFluor-594 and AlexaFluor-488 conjugated cholera toxin B (CTb) (Life Technology) were unilaterally infused at a rate of 0.1 μl/min for 4 min (0.4 μl total volume; 5 μg/μl) into BA and PL, respectively. The injectors remained in the injection sites for 10 min to allow for the diffusion of CTb. Rats were placed back in their home cages to allow for 1 week of post-operative recovery.

### Behavioral procedures

Twenty-six rats were randomly assigned to three different groups: DIFF (n = 10), SAME (n = 10) and HOME (n = 6). We used a three-context (“ABC”) renewal procedure^17^ in which one group (DIFF) was conditioned in context A, extinguished in context B and tested in context C and another group (SAME) was conditioned in context A, extinguished in context C and tested in context C. Rats in the HOME control group were conditioned in context A and extinguished either in context B or C (counterbalanced); they were left in their home cages during the test session. Unlike an ABA design, the ABC design permits the assessment of fear and Fos expression to an extinguished CS independent of fear to the context in which the CS is tested. That is, the test context (context C) has never been paired with footshock. Moreover, this design allowed us to test all rats in the identical context, hence any differences in behavior or c-Fos expression can be attributed to the meaning of the CS in that context and not the CS or context itself.

After surgery, rats underwent fear conditioning in context A. Conditioning consisted of five tone (CS; 10 s, 80 dB, 2 kHz) -footshock (US; 1.0 mA, 2 s) pairings with 60 s intertrial intervals (ITIs). Twenty-four hours after the conditioning session, rats underwent fear extinction in context B or context C in which they received 45 tone-alone (10 s, 80 dB, 2 kHz, 30 s ITI) presentations. Prior to the extinction session, rats were exposed to the alternative context (i.e., they were exposed to context C if they were extinguished in context B) to ensure that the test contexts were equally familiar for all of the rats. Twenty-four hours after extinction, rats were returned to context C for a test session consisting of five tone-alone (10 s, 80 dB, 2 kHz, 30 s ITI) presentations. In all behavioral sessions, the chamber position of each animal was counterbalanced.

### Immunohistochemistry

Ninety minutes after the first tone of the retrieval test session, rats were overdosed with sodium pentobarbital (0.5 ml) and were transcardially perfused with ice-cold 0.01 M PBS (pH 7.4) and 4% paraformaldehyde (PFA) in 0.1 M phosphate buffer (pH 7.4). Animals were sacrificed in groups of three, with one rat from each group represented in each squad (one rat from the HOME control group was randomly chosen to be sacrificed along with the SAME and DIFF rats in each squad). Brains were extracted and stored in 4% PFA solution for 18 h at 4°C and transferred to 30% sucrose at 4°C. Coronal brain sections (30 μm) were made on a cryostat maintained at −20°C. Sections containing vCA1 and vSUB were collected every 210 μm.

Immunohistochemistry was performed on free-floating brain sections. The sections were washed two times in 1 × Tris-buffered saline (TBS, pH 7.4) for 10 min followed by a third wash in 1 × TBS with 0.1% Tween 20 (TBST, pH 7.4). Brain sections were then incubated in 10% normal donkey serum (NDS) in TBST for 1 h at room temperature followed by two washes in TBST for 5 min. Tissue was then incubated in primary antibody solution in TBST with 2% NDS (goat anti-c-Fos antibody at 1:2000; sc-52-G, Santa Cruz Biotechnology) for 72 h at 4°C. Brain sections were then washed three times in TBST for 10 min and were incubated in secondary antibody solution in TBST with 2% NDS (biotinylated donkey anti-goat secondary antibody at 1:200; sc-2042, Santa Cruz Biotechnology) for 2 h at room temperature. After being rinsed in TBST three times for 10 min, brain sections were incubated in streptavidin conjugated AlexaFluor 350 (Streptavidin-AF350 at 1:500; s-11249, Life Technology) in TBST with 2% NDS for 1 h at room temperature. Tissue was washed three times in TBS for 10 min and then was mounted onto subbed slides in 0.9% saline and cover slipped with Fluoromount (Sigma-Aldrich).

### Image analysis

Three images for the vCA1 (−5.6, −6.3, and −6.8 mm posterior to bregma) and two images for the vSUB (−6.3, and −6.8 mm posterior to bregma) were taken for the quantification. All images were taken at 10 × -magnification (660 μm × 876 μm, 0.58 mm^2^) with an Olympus BX53 microscope. For each region, single-, double-, and triple-labeled neurons for each fluorophore were counted. Counts for each image of the brain region were averaged.

### Histology

After perfusion, coronal sections (40 μm) were collected on a cryostat (−20°C) and mounted onto subbed slides to confirm the CTb placements.

### Data analysis

All data were analyzed with analysis of variance (ANOVA). Post-hoc comparisons in the form of Fisher's protected least significant difference (PLSD) post hoc tests, which were performed after a significant overall F ratio. All data are represented as means ± SEM. Four rats failed to extinguish (freezing during the last block was >80% of the freezing in the first block) and another two rats exhibited Fos counts that were >2.5 SD outside the group mean; these rats were excluded from the neuronal and behavioral analyses. Hence, the final group sizes were SAME (*n* = 8), DIFF (*n* = 8), and HOME (*n* = 4).

## Author Contributions

Both J.J. and S.M. analyzed data, contributed to the writing the manuscript, and reviewed the completed manuscript. S.M. designed the experiments and J.J. conducted all of the experimental procedures.
